# Prevalence of Sinus Mucosal Abnormalities on CT of the Head Performed for Headache When Compared With Those Performed for Other Indications

**DOI:** 10.7759/cureus.56608

**Published:** 2024-03-20

**Authors:** Sadhana Kalidindi, Sanjay Gandhi

**Affiliations:** 1 Radiology, University of Bristol, Bristol, GBR; 2 Radiology, North Bristol Hospitals NHS Trust, Bristol, GBR

**Keywords:** sinus thickening, lm score, sinusitis, headache, ct head

## Abstract

Background

There is a high prevalence of mucosal abnormalities of paranasal sinuses on CT Head scans performed for all indications. The purpose of this study is to see whether or not such abnormalities are more common in scans performed on patients presenting with headaches when compared with those without headaches.

Methods

Images of CT scans of the brain of 100 consecutive patients from each of the two study groups (a total of 200 scans) were retrospectively reviewed for the presence of sinus mucosal abnormalities and their Lund-Mackay (LM) scores were calculated. A corrected LM score was also calculated using a correction factor for non-visualized sinuses in some scans and osteomeatal complexes in all scans. Radiological reports for these scans were also reviewed to note whether or not they contained any comments on the sinuses. All the reviewed scans were performed between January 1, 2021 and January 22, 2021.

Results

In the headache group, 17 patients had an LM score above 4 (which was used as the main cut-off point for this study). In the non-headache group, 16 patients had a score greater than 4. The mean LM score in the headache group was 1.24 and in the non-headache group was 1.4. There has been no significant difference in the comparison when corrected LM scores were used. In the headache group, 22 radiology reports contained comments on the sinuses compared to 11 reports in the non-headache group.

Conclusion

Results of this study indicate that there is no significant difference in the prevalence of clinically important sinus mucosal abnormalities in patients who had a brain CT for headache when compared with other indications. It was found that radiologists tend to comment on the sinuses more often when the indication was headache. It may be reasonable for radiologists to consider reviewing this practice. This might reduce unnecessary referrals to ENT and, more importantly, avoid missing other reasons for headaches.

## Introduction

There is a high prevalence of sinus mucosal abnormalities detected incidentally on CT scans of the Head with some studies reporting rates as high as 42.5% in asymptomatic adults [1]. A CT head is used to investigate headache especially when it is of abrupt or acute onset [2]. Also, headache is a common complaint for patients presenting in Accident and Emergency Departments and nearly 15% of patients from these departments are referred for a CT Head as one of the investigations [3].

It is, therefore, common for radiologists to encounter findings of sinus mucosal abnormalities on CT scans performed for headaches and to face a dilemma about whether or not these findings need to be mentioned in the report. When reported, further dilemmas might ensue within the referring clinical team about whether or not they are clinically significant and relevant to the patient’s symptoms. Assuming that the sinus findings are the cause of a patient’s headache can lead to misdiagnosis [3].

The purpose of this study is to see if there is any difference in the prevalence and severity of sinus mucosal findings on CT Heads performed for headaches when compared with a similar number of scans in a non-headache group. The observations from this study could be of value to radiologists in making an informed decision on whether and when they should comment on the presence or absence of sinus findings on head CT in patients with headaches.

Several systems have been developed to stage sinus findings on CT. These include Kennedy, Levine, and May, Harvard, and Lund-Mackay (LM) systems [4]. Out of these scores, the LM score is considered to be simple and is shown to have high interobserver reliability and correlate well with disease severity. For this study, we used the LM score to compare both groups. We used a cut-off of 4 as the main point which was described as the minimum score to indicate clinically significant disease that might need treatment [5].

## Materials and methods

We performed a retrospective review of images and reports of 200 non-contrast CT Head scans performed at Southmead Hospital, North Bristol NHS Trust, United Kingdom, between January 1, 2021 and January 22, 2021. This study followed the University of Bristol and North Bristol NHS Trust’s ethics committee guidelines for a retrospective review.

The study group included 100 consecutive patients who had a CT of the Head for Headache and another 100 consecutive patients who had it for an indication other than headache. Patients with a history of trauma were excluded from the study. The review also included radiological reports of these 200 patients.

All images were reviewed on the Picture archiving and communication system (PACS). All the available series were reviewed with windowing as required. The radiological reports were reviewed on the Radiology Information System (RIS). Other information like the patient demographics, source of referral, and indication for the study were also obtained from RIS.

Each study was evaluated for the presence of any mucosal abnormality in the frontal, maxillary, anterior ethmoid, posterior ethmoid, and sphenoid sinuses on each side. Each sinus was graded as either normal, partially opacified, or totally opacified. Sinuses with small polyps were included in the partially opacified category and those with large ones are classified as totally opacified.

A score of 0 was assigned to each normal sinus, a score of 1 for each partially opacified sinus, and a score of 2 for each totally opacified sinus. The score was 0 for osteomeatal complexes as these could not be scored on these standard brain CT scans. An LM score was calculated for each patient based on this evaluation.

In addition to the calculated LM score as obtained above, a “corrected LM score” was calculated for all patients using the method described by Nazri et al. [4]. The correction factor applied was based on the number of partially or totally unseen sinuses on each scan. The osteomeatal complexes were considered totally unseen. The correction factor used was 1 for a partially unseen sinus and 2 for a totally unseen sinus.

## Results

Demographic data and source of referral

The mean age for the whole group of 200 patients was 61 years (SD 21). The mean age for the headache group was 52.8 years (SD 20). For the non-headache group, the mean age was 69.25 years (SD 19). In the headache group, there were 42 males and 58 females. In the non-headache group, the number of males was 51 and females was 49.

As shown in Table 1, more than 50% of referrals in all groups were from the A&E department. Out of the remainder, inpatients were the next common source followed by GPs and outpatients. This pattern is similar in both groups.

**Table 1:** Demographics and origin of the referral

	Whole Group (n=200)	Headache Group (n=100)	Non-Headache Group (n=100)
Youngest patient	19 years	19 years	23 years
Oldest Patient	101 years	92 years	101 years
Mean Age	61 years	52.8 years	69.25 years
SD	21	20	19
Males	93	42	51
Females	107	58	49
A&E Referral	114	59	55
GP Referral	22	13	09
IP Referral	49	20	29
OP Referral	13	07	07
Unknown	2	01	00

Analysis of the sinus abnormalities

In both groups, ethmoid sinuses showed the highest rate of mucosal abnormality. This was followed by the maxillary sinuses. Frontal sinuses were the least commonly involved. The pattern was somewhat similar for both groups. Table 2 compares the sinus mucosal abnormalities in both groups.

**Table 2:** Comparative analysis of sinus mucosal abnormalities

	Whole Group (n=200)	Headache Group (n=100)	Non-Headache Group (n=200)
Rt Frontal			
Normal	194	97	97
Abnormal	4	2	2
Not seen	2	1	1
Rt Maxillary			
Normal	167	83	83
Abnormal	32	15	17
Not seen	1	2	0
Rt Ant Ethmoid			
Normal	161	84	77
Abnormal	39	16	23
Rt Post Ethmoid			
Normal	159	82	78
Abnormal	41	18	22
Rt Sphenoid			
Normal	184	93	91
Abnormal	16	7	9
Lt Frontal			
Normal	193	97	96
Abnormal	4	1	3
Not seen	3	2	1
Lt Maxillary			
Normal	168	84	84
Abnormal	30	14	16
Not seen	2	2	0
Lt Ant Ethmoid			
Normal	154	81	73
Abnormal	46	19	27
Lt Post Ethmoid			
Normal	163	83	80
Abnormal	37	17	20
Lt Sphenoid			
Normal	180	95	85
Abnormal	20	5	15

There was no evidence of any increased prevalence of mucosal abnormalities in any of the sinuses in the headache group when compared with the non-headache group. On the other hand, in this study group, there was a mildly increased prevalence of mucosal abnormalities in the ethmoid sinuses and the left sphenoid sinus within the non-headache group.

The LM score (calculated and corrected)

LM scores were calculated for all patients and corrected LM scores were also generated. The correction factor was based on the non-scoring status of OM complexes in all patients and the number of other partially or totally unseen sinuses. 

Both groups were compared using several LM score cut-off points (above which the patients could be considered as having sinusitis). Table 3 shows the number of patients above various cut-off points in the headache group compared with those in the non-headache group. A comparison between the two groups was also made using the mean LM scores (Table 4).

**Table 3:** Number of patients with LM scores above various cut-off points

	Headache Group (n=100)	Non-Headache Group (n=100)
LM Score Cut off		
Above 2	24	35
Above 3	19	25
Above 4	17	16
Above 5	11	12

**Table 4:** LM score comparison (mean and SD)

	Headache Group	Non-Headache Group
LM Score		
Range	0 - 9	0 - 10
Mean	1.23	1.43
SD	1.99	2.10

There was no significant difference in any of the comparative analyses if corrected LM scores were used instead of calculated LM scores. So, these were not represented in the results presented here.

When lower cut-off points are used (above which the mucosal abnormality was considered to be potentially clinically significant), there were more abnormal scans in the non-headache group than in the headache group. At the higher cut-off points (4 and 5), the number was similar in both groups. There was no statistically significant difference in the Mean LM scores between the headache and non-headache groups.

The radiological reports of all patients were reviewed to see if comments were made on the paranasal sinuses. Out of the total 200 reports, 33 contained comments about the sinuses whereas 167 reports did not include any relevant comments. Table 5 compares the mention of sinuses within reports for the headache group vs the non-headache group.

**Table 5:** Mention of imaging observations related to sinuses in radiology reports

	Headache Group	Non-Headache Group
Sinuses Reported		
Yes	22	11
No	78	89

The reporting radiologists commented on the sinuses more often when the indication was headache (22%) when compared with scans done for non-headache indications (11%).

## Discussion

Even when trauma is excluded, CT Head remains the investigation of choice for headaches of acute onset. Referrals from Accident and Emergency departments for patients with acute atraumatic headaches are one of the main sources for such scans followed by referrals from inpatients, GPs, and outpatients.

There is a very high prevalence of incidental sinus mucosal abnormalities in the general population, which will be seen in CT Head scans performed for any indication. This will result in a significant number of scans performed for atraumatic headaches showing features that could be interpreted as “sinusitis.” As shown in this study, radiologists could be twice more likely to comment on the findings in the sinuses when the indication for the CT Head was headache. 

Mention of the presence of sinus mucosal abnormality in a CT scan report in a patient with atraumatic headache could lead to a clinical misdiagnosis of sinusitis being the cause of the patient’s symptoms [6]. It has been reported that in several cases where there was a misdiagnosis of subarachnoid hemorrhage, a diagnosis of “sinusitis” was made instead [7]. 

Also, studies have shown that findings of sinus mucosal abnormalities should not be used to predict that they are the cause of the patient’s symptoms or localize areas of facial pain or pressure [8]. It has also been reported that there is no statistically significant association between the extent and stage of CT findings and the severity of the patient’s symptoms [9].

In this study, we set out to see if there is any reason to justify or warrant a mention of the presence of sinus mucosal abnormalities in patients who had a CT of the Head for atraumatic headache. To do this we compared not only the prevalence of sinus findings in each group but also the stage of those findings using the LM scoring.

The results of the analysis of this relatively large study group containing 100 patients who had CT Heads for atraumatic headache and a control group of 100 patients who had no headache have shown that there is no evidence of any increased prevalence of sinus mucosal abnormalities in the headache group. The sinus findings and their distribution were largely similar between the two groups. In addition, this study also shows that there is no significant difference in the grade of the disease amongst the sinus-positive scans across both groups, as demonstrated by the comparative evaluation of the LM scores.

Based on these observations, it appears reasonable to recommend that radiologists review their current practice and consider not reporting sinus mucosal abnormalities detected on CT head scans performed for atraumatic headaches and not making any conclusions about sinusitis. The exception to this would be patients where the clinical diagnosis is suspected rhinosinusitis or when there is extensive opacification of multiple sinuses. 

Some radiologists may feel strongly about reporting all abnormalities including sinus mucosal thickening. Therefore, another alternative would be to add a caveat to CT reports that sinus mucosal abnormalities are common and might not be the actual cause of the patient’s symptoms.

## Conclusions

This study included a comparison of sinus mucosal findings on a relatively large number of CT Head scans performed for atraumatic headaches with a control group of an equal number of CT Heads performed for other non-headache indications. In this study group, it is evident that there is no evidence of any increased prevalence of mucosal thickening or opacification of the sinuses in the headache group when compared to the control group. There was also no evidence of an increased number of more advanced-stage mucosal abnormalities based on the LM score. In view of these observations, it may be reasonable for radiologists to review their current practice and consider not reporting sinus mucosal abnormalities on CT Heads performed for an atraumatic headache unless there is a specific clinical suspicion for rhinosinusitis or the opacification is complete or extensive and involves multiple sinuses. This might prevent potential misdiagnosis and missing other reasons for headaches in these patients.
